# Supplementary figures and images for: Temporomandibular joint disorders in skeletal class II patients referred to orthognathic surgery: A cross-sectional study
Source: PLoS One. 2024 Feb 15;19(2):e0297944. doi: 10.1371/journal.pone.0297944 (PMC10868827; doi:10.1371/journal.pone.0297944)

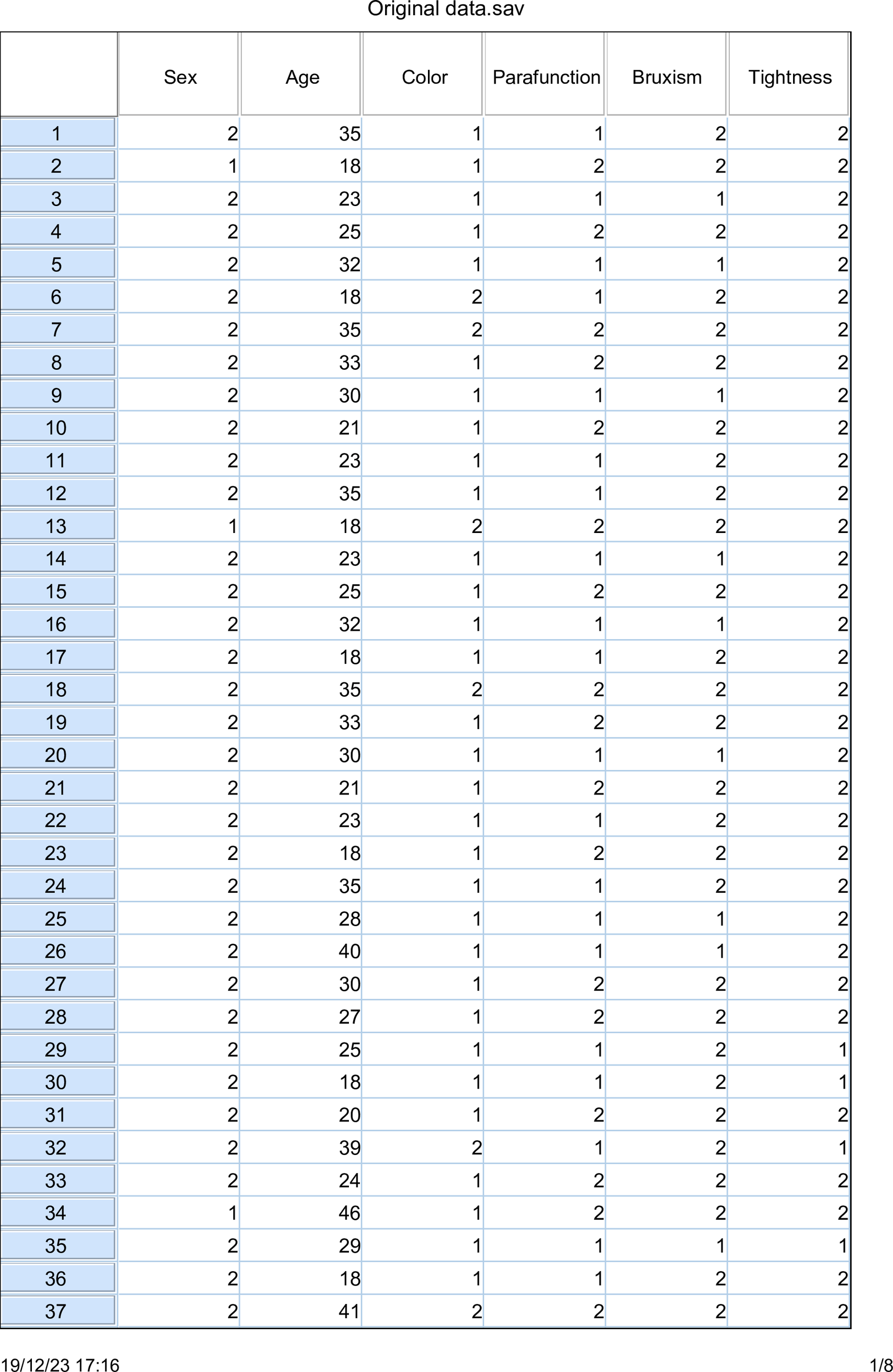

Supplement: S1 Dataset — (TIF) [file pone.0297944.s001.tif]

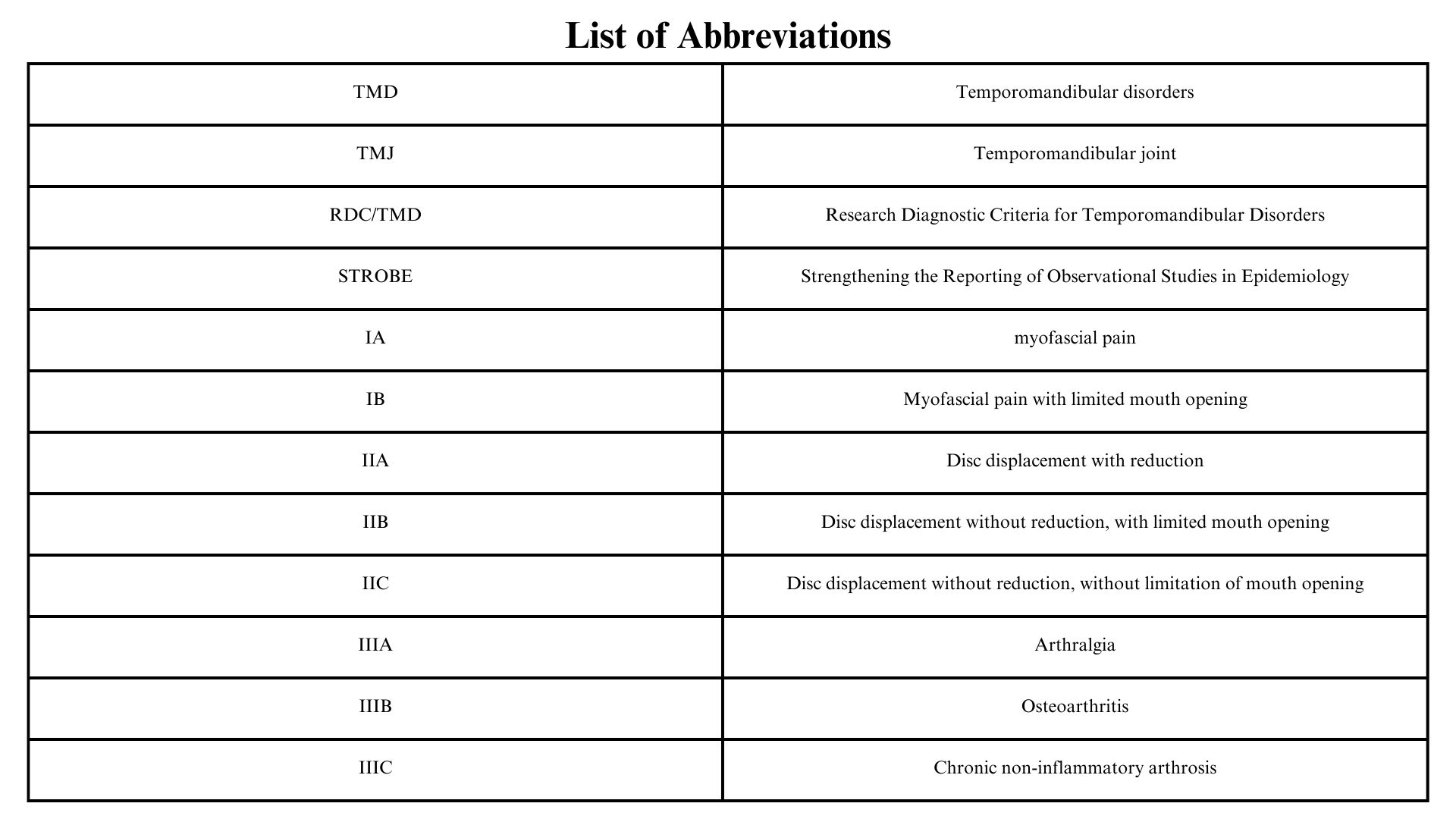

Supplement: S1 Table — (TIF) [file pone.0297944.s002.tif]

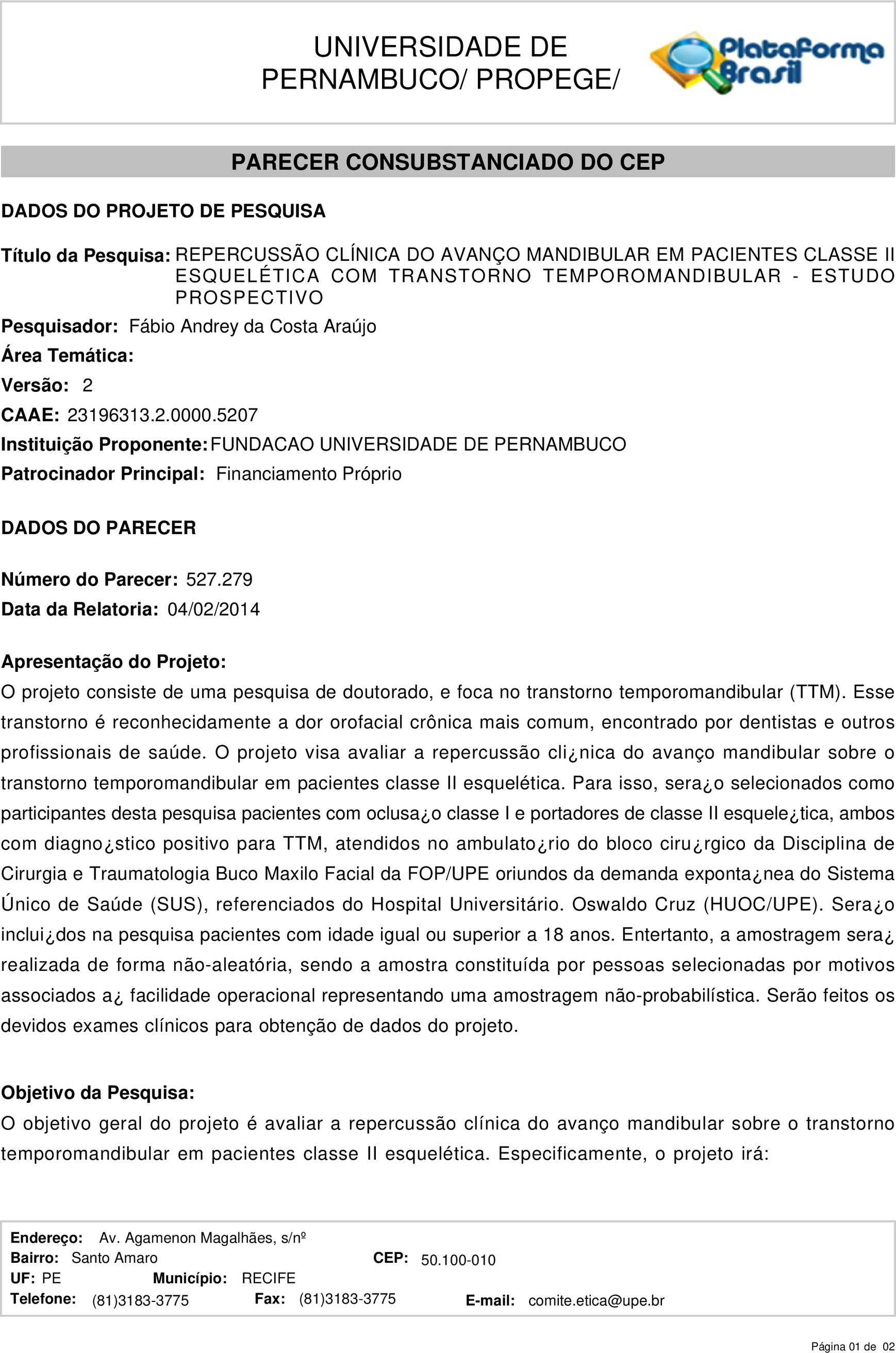

Supplement: S1 Appendix — (TIF) [file pone.0297944.s003.tif]

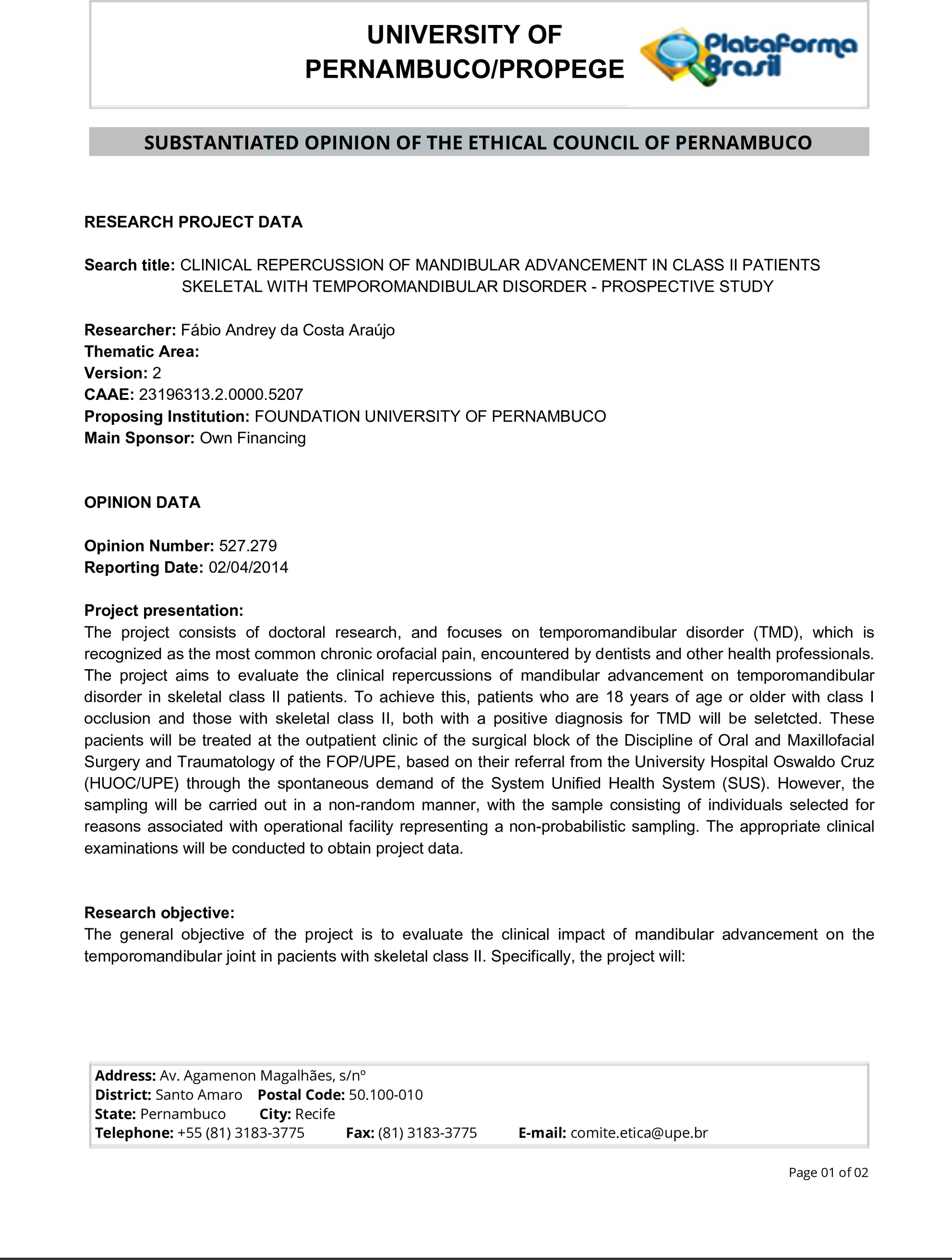

Supplement: S2 Appendix — (TIF) [file pone.0297944.s004.tif]
