# Supplementary material for: Temporomandibular joint disorders in skeletal class II patients referred to orthognathic surgery: A cross-sectional study
Source: PLoS One. 2024 Feb 15;19(2):e0297944. doi: 10.1371/journal.pone.0297944 (PMC10868827; doi:10.1371/journal.pone.0297944)
Supplement: Attachment — Submitted filename: Response to Reviewers.docx [file pone.0297944.s005.docx]

Bárbara Araújo da Silva

R. Arnóbio Marquês, 310,

Recife, Brazil 50.100-130.

E-mail: barbara.araujosilva@upe.br

December 19, 2023

To the Editorial Board of the PLOS ONE.

Dear Editor,

On behalf of all the authors, I would like to thank you for the opportunity to clarify the remarks made in our manuscript entitled “*Temporomandibular joint disorders in skeletal Class II patients referred to orthognathic surgery: A cross-sectional study*”. We addressed the suggestions raised by the associate editor and reviewers and generated the revised version of the manuscript to be considered for publication as a publication as a Research Article in this prestigious Journal.

All the changes we have made and the responses to editor and reviewer questions are listed below and highlighted in blue in the manuscript:

**JOURNAL REQUIREMENTS**

**1. Please ensure that your manuscript meets PLOS ONE's style requirements, including those for file naming.**

**Answer:** Thank you for pointing out this issue, we have now corrected this.

**2. In your Data Availability statement, you have not specified where the minimal data set underlying the results described in your manuscript can be found. PLOS defines a study's minimal data set as the underlying data used to reach the conclusions drawn in the manuscript and any additional data required to replicate the reported study findings in their entirety. All PLOS journals require that the minimal data set be made fully available. For more information about our data policy, please see** [**http://journals.plos.org/plosone/s/data-availability**](http://journals.plos.org/plosone/s/data-availability)**.**

**Upon re-submitting your revised manuscript, please upload your study’s minimal underlying data set as either Supporting Information files or to a stable, public repository and include the relevant URLs, DOIs, or accession numbers within your revised cover letter.**

**Answer:** Thank you for pointing out this issue, we have now added in the supporting files the data used to set our results.

**3. Your ethics statement should only appear in the Methods section of your manuscript. If your ethics statement is written in any section besides the Methods, please move it to the Methods section and delete it from any other section. Please ensure that your ethics statement is included in your manuscript, as the ethics statement entered into the online submission form will not be published alongside your manuscript.**

**Answer:** Thank you for pointing out this issue, we have now corrected this.

**4. Please include captions for your Supporting Information files at the end of your manuscript, and update any in-text citations to match accordingly.**

**Answer:** Thank you for pointing out this issue, we have now included after the references a

section of Supporting Information.

**REVIEWERS CONSIDERATIONS**

**REVIEWER 1:**

1. **Please be sure to use only keywords accordingly to medical subject headings (Mesh word) for a better indexing.**

**Answer:** The keywords used were all taken from MESH, see below MeSH unique ID of them:

Temporomandibular Joint - D013704

Temporomandibular Joint Disorders - D013705

Orthognathic Surgery - D057909

Retrognathia - D063173

Malocclusion, Angle Class II - D008312

2. **I suggest you add a table with the list of abbreviations used in the text.**

**Answer:** As suggested, we add a table with the list of abbreviations in the supporting files.

3. **I suggest you implement the abstract in order to make it more understandable to authors.**

**Answer:** As suggested, the Abstract was improved.

4. **The introduction should be expanded perhaps by adding a section on temporomandibular disorders.**

**Answer:** As suggested, the section of Introduction was improved and expanded.

**REVIEWER 2:**

1. **The introduction should stress the different causes of TMD and possible linkage with other systemic disease such as vitamin D deficiency. Moreover, I suggest, in order to have a complete framework of the disease, to add a brief overview of the several therapeutical conservative approaches to TMD.**

**Answer:** As suggested, we added different causes of TMD and made an overview of the

conservative approaches to TMD in the section Introduction.

2. **Have the patients underwent prior treatment fo TMD? please, specify this point.**

**Answer:** Thank you for pointing out this issue. Some of the patients underwent prior treatment for TMD, however, at the moment of the research none of them were doing any kind of treatment for the last six months. We have now clarified this point in the section Results.

3. **Please, replace DTM with TMD at line 199. Moreover, I suggest to add the clinical implication of your findings. The authors state that the prevalence of TMD in skeletal II is 47% versus the 65% of the general population, but in conclusion affirm that "Patients with skeletal Class II dentofacial deformity who were referred for orthognathic surgery showed higher prevalence of TMD than expected for the general population". please, replace the sentence**

**Answer:** Thank you for pointing out this issue, we have now corrected this.

I reinforce that the research meets all applicable standards with regard to the ethics and research integrity. I declare that this manuscript, or any part of it, has not been submitted or published and will not be submitted elsewhere for publication while being considered by your journal. Furthermore, all authors have no conflict of interest to declare as well as approved the manuscript and agreed with its submission to this Journal.

Thank you for your consideration!

Kind regards,

**Corresponding author:**

Bárbara Araújo da Silva
